# DNABERT2-CAMP: A Hybrid Transformer-CNN Model for *E. coli* Promoter Recognition

**DOI:** 10.3390/genes17010027

**Published:** 2025-12-28

**Authors:** Hua-Lin Xu, Xiu-Jun Gong, Hua Yu, Ying-Kai Wang

**Affiliations:** 1 Department of Intelligent Technology, Tianjin Polytechnic University, Tianjin 300340, China; xuhualin@pctj.edu.cn; 2 School of Artificial Intelligence, Tianjin University, No. 135 Yaguan Road, Haihe Education Park, Tianjin 300354, China; 18322461810@163.com; 3 Key Laboratory of Systems Bioengineering (Ministry of Education), No. 135 Yaguan Road, Haihe Education Park, Tianjin 300354, China; 4 School of Computer Science and Technology, Tianjin University, No. 135 Yaguan Road, Haihe Education Park, Tianjin 300354, China; yuhua@tju.edu.cn

**Keywords:** *E. coli*, promoter recognition, DNABERT2, convolutional neural network, transformer

## Abstract

Background: Accurate recognition of promoter sequences in *Escherichia coli* is fundamental for understanding gene regulation and engineering synthetic biological systems. However, existing computational methods struggle to simultaneously model long-range genomic dependencies and fine-grained local motifs, particularly the degenerate −10 and −35 elements of σ70 promoters. To address this gap, we propose DNABERT2-CAMP, a novel hybrid deep learning framework designed to integrate global contextual understanding with high-resolution local motif detection for robust promoter identification. Methods: We constructed a balanced dataset of 8720 experimentally validated and negative 81-bp sequences from RegulonDB, literature, and the *E. coli* K-12 genome. Our model combines a pre-trained DNABERT-2 Transformer for global sequence encoding with a custom CAMP module (CNN-Attention-Mean Pooling) for local feature refinement. We evaluated performance using 5-fold cross-validation and an independent external test set, reporting standard metrics including accuracy, ROC AUC, and Matthews correlation coefficient (MCC). Results: DNABERT2-CAMP achieved 93.10% accuracy and 97.28% ROC AUC in cross-validation, outperforming existing methods including DNABERT. On an independent test set, it maintained strong generalization (89.83% accuracy, 92.79% ROC AUC). Interpretability analyses confirmed biologically plausible attention over canonical promoter regions and CNN-identified AT-rich/-35-like motifs. Conclusions: DNABERT2-CAMP demonstrates that synergistically combining pre-trained Transformers with convolutional motif detection significantly improves promoter recognition accuracy and interpretability. This framework offers a powerful, generalizable tool for genomic annotation and synthetic biology applications.

## 1. Introduction

The precise identification of *E. coli* promoters is essential for advancing synthetic biology, as predictable control of gene expression forms the foundation for designing genetic circuits, biosensors, and engineered microbial systems [1]. As the primary model organism in both molecular and synthetic biology, *E. coli* primarily utilizes σ70-dependent promoters—characterized by conserved −10 (TATAAT) and −35 (TTGACA) motifs—to initiate transcription [2]. Accurate recognition of these regulatory sequences not only supports the reconstruction of native gene regulatory networks [3] but also enables the rational design and optimization of synthetic promoters for customized gene expression programs [4,5].

### 1.1. Traditional Approaches

Early computational methods for promoter recognition relied heavily on sequence conservation analysis. Position weight matrices (PWMs) [6] and hidden Markov models (HMMs) [7] are widely applied to capture the statistical properties of conserved motifs, such as the −10 and −35 boxes. While effective for highly conserved σ70 promoters, these methods assumed positional independence. They struggled with non-typical promoters or variable spacer lengths, often leading to high false-positive rates in complex genomic backgrounds.

### 1.2. Machine Learning Methods

With the advent of machine learning, classifiers such as support vector machines (SVMs) and random forests (RFs) have been introduced for promoter recognition tasks. These models extracted handcrafted features, such as k-mer frequencies, GC content, and structural properties of DNA [6]. Although they improved performance compared to PWMs and HMMs, their effectiveness was constrained by the quality of feature engineering and their limited ability to capture long-range dependencies between regulatory motifs.

### 1.3. Deep Learning Advances

Deep learning methods have further advanced promoter recognition by enabling automatic feature extraction from raw DNA sequences. Convolutional neural networks (CNNs) demonstrate a strong capability in detecting local sequence motifs, including the conserved −10 and −35 boxes [8]. Recurrent neural networks (RNNs), particularly long short-term memory (LSTM) networks, are applied to capture sequential dependencies in longer DNA fragments [9]. However, CNNs are limited in modeling long-range dependencies, while RNNs suffer from vanishing gradients [10] and high computational costs, which restrict their scalability to genome-wide applications.

### 1.4. Transformer-Based Models

Recently, Transformer-based architectures have revolutionized biological sequence analysis, establishing themselves as a powerful paradigm for genomic tasks. DNABERT, a pre-trained Bidirectional Encoder Representations from Transformers (BERT) model adapted for DNA sequences, introduces k-mer tokenization and self-attention mechanisms to capture global contextual information [11]. DNABERT significantly improved promoter recognition and transcription factor binding site prediction, reducing reliance on handcrafted features. Nevertheless, existing Transformer-based models still face challenges in balancing sensitivity and specificity, handling imbalanced datasets, and recognizing non-typical promoters.

### 1.5. Research Gap and Motivation

While the aforementioned approaches each represent significant advances, a critical synthesis reveals a persistent and complementary challenge that limits their efficacy for comprehensive promoter recognition. Traditional methods (PWMs, HMMs) are fundamentally constrained by assumptions of positional independence. Classical machine learning models (SVMs, RFs) remain dependent on manual feature engineering and struggle to model long-range dependencies. Deep learning architectures offer automated feature learning but present a trade-off: CNNs excel at local motif detection yet are limited in modeling long-range genomic context, while RNNs can capture sequence dependencies but face challenges with computational efficiency and gradient propagation. More recently, Transformer-based models like DNABERT have demonstrated remarkable capability in capturing global sequence semantics through self-attention, yet they may lack the granularity for precise, localized motif resolution, particularly for atypical or weakly conserved promoter classes.

This dichotomy between global contextual understanding and localized motif sensitivity creates a significant gap in the current methodological landscape. Consequently, there is a pressing need for a novel computational framework capable of synergistically integrating these two complementary capabilities: harnessing global context for understanding sequence architecture while simultaneously achieving precise detection of conserved regulatory motifs. Addressing this need is crucial for advancing the accuracy, robustness, and biological interpretability of promoter identification, especially in complex genomic applications.

### 1.6. Contributions of This Study

To address these limitations, we propose DNABERT2-CAMP, a hybrid deep learning framework that integrates a pre-trained DNABERT-2 Transformer with a custom CAMP module (CNN-Attention-Mean Pooling) for enhanced feature extraction and robust classification. The main contributions of this work are as follows:

(a) We introduce a novel hybrid architecture that effectively combines the global contextual modeling capabilities of DNABERT-2 with the local motif detection strengths of convolutional networks, enabling comprehensive analysis of both long-range dependencies and precise local patterns in promoter sequences.
(b) Our model achieves state-of-the-art performance with a ROC AUC of 97.28% and accuracy of 93.10% in 5-fold cross-validation, significantly outperforming existing methods while maintaining robust generalization on independent test sets.
(c) We develop an innovative feature fusion strategy that integrates global sequence embeddings with locally refined features, creating a biologically grounded representation that effectively handles both typical σ70 promoters and challenging non-typical promoters for practical applications in synthetic biology.

This study highlights the potential of integrating pre-trained language models with CNNs for genomic sequence analysis, offering both high predictive accuracy and biological interpretability.

## 2. Literature Review

The computational recognition of promoters has undergone significant methodological evolution, driven by advances in both biological understanding and machine learning techniques. This section provides a systematic overview of this progression, delineating the core principles, representative advancements, and inherent limitations of each major paradigm. This historical and technical context is essential for positioning the proposed DNABERT2-CAMP model within the broader research landscape and clarifying the specific gap it aims to address.

### 2.1. Early Rule-Based and Consensus-Driven Methods

Initial computational efforts were fundamentally rule-based, focusing on identifying promoters through consensus sequences and simple statistical models. The highly conserved −10 (TATAAT) and −35 (TTGACA) boxes of σ70 promoters served as primary targets. Techniques such as consensus scanning and early implementations of Position Weight Matrices (PWMs) quantified nucleotide preferences at fixed positions, operating under the assumption of positional independence [6]. While effective for identifying canonical, strong promoters, these methods exhibited poor sensitivity to sequence context, variable spacer lengths, and degenerate or non-canonical motifs, leading to high false-positive rates in complex genomic backgrounds [12].

The introduction of Hidden Markov Models (HMMs) offered a more flexible, probabilistic framework capable of modeling variable-length spacers and simple state dependencies between motif sites [7]. This represented an improvement over rigid PWMs. However, HMMs still required carefully curated training datasets and struggled to capture the complex, long-range nucleotide interactions or the highly degenerate motifs characteristic of promoters recognized by alternative σ factors.

### 2.2. Feature-Engineered Machine Learning Approaches

The field shifted towards data-driven classification with the adoption of machine learning. Algorithms such as Support Vector Machines (SVMs) and Random Forests (RFs) became prevalent, utilizing a diverse set of handcrafted sequence features. These included k-mer frequencies, GC content, DNA structural properties (e.g., duplex stability [13], bendability), and Z-curve coordinates [14]. By incorporating a broader spectrum of sequence attributes, these methods generally achieved higher accuracy than consensus-based techniques.

A critical limitation of this paradigm was its heavy reliance on feature engineering—the manual selection and transformation of sequence properties into a numerical representation suitable for classifiers. This process not only required substantial domain expertise but also inherently constrained the model’s capacity to automatically discover novel or complex higher-order patterns. Furthermore, these approaches typically failed to adequately model long-range dependencies between spatially separated regulatory elements, a key shortcoming for accurately recognizing promoters where the precise arrangement and interaction of motifs are crucial.

### 2.3. Deep Learning for Automated Feature Learning

Deep learning revolutionized the field by enabling end-to-end learning directly from raw nucleotide sequences, thereby automating feature extraction. Convolutional Neural Networks (CNNs) emerged as a particularly powerful architecture for detecting localized, conserved motifs without prior knowledge [8]. Models such as iPromoter-BnCNN [15] and pcPromoter-CNN [16] employed multiple convolutional filters to scan for patterns resembling the −10 and −35 boxes, demonstrating superior performance over traditional SVM-based models.

To address the sequential nature of DNA, Recurrent Neural Networks (RNNs), and particularly Long Short-Term Memory (LSTM) networks, were introduced to capture dependencies across longer sequence contexts [9]. While conceptually well-suited, LSTMs proved computationally expensive for long sequences and were prone to issues like vanishing gradients, limiting their practical scalability for genome-wide analyses [10].

A significant conceptual limitation of these early deep learning architectures was their specialization: CNNs excelled at local motif detection but were inherently weak in modeling global, intra-sequence relationships due to their limited receptive fields. RNNs could, in theory, handle longer contexts but were often inefficient and unstable in practice. Hybrid or ensemble models like MULTiPly [17] attempted to combine strengths but often still relied on pre-defined input representations rather than learning fully contextualized embeddings from the data.

### 2.4. The Rise of Pre-Trained Transformer Models in Genomics

Inspired by breakthroughs in natural language processing (NLP), Transformer-based models adapted the self-attention mechanism to biological sequences, marking a paradigm shift. DNABERT [11] pioneered this approach by pre-training on large-scale, unlabeled genomic corpora using a masked language modeling (MLM) objective with k-mer tokenization. This process allowed the model to learn rich, contextualized embeddings that captured both local “syntax” and global “semantics” of DNA sequences. DNABERT and similar models achieved state-of-the-art results in promoter recognition and related tasks, significantly reducing reliance on task-specific feature engineering and demonstrating remarkable transfer learning capabilities.

Subsequent variants, including DNABERT-2, refined the architecture, training objectives, and pre-training data, further enhancing generalization across diverse genomic prediction tasks. Despite their strengths, purely Transformer-based approaches can sometimes lack the high-resolution, motif-specific sensitivity that is a hallmark of CNNs. The global context provided by self-attention is powerful, but it may not always match the precision of convolutional filters in pinpointing subtle, highly conserved local patterns that are critical for biological function.

### 2.5. Hybrid Architectures and Remaining Challenges

Recognizing the complementary strengths of CNNs and Transformers, recent research has explored hybrid architectures. Models combining CNN layers with attention mechanisms or using Transformers to augment CNN-extracted features have been applied to various bioinformatics tasks, including recent promoter recognition studies [18]. However, many existing hybrids either employ Transformers as mere feature augmentors without deep integration or lack a principled, optimized fusion mechanism to dynamically balance global context with local detail.

A critical synthesis of the literature reveals a persistent methodological gap: no single architecture yet seamlessly and effectively integrates the global contextual understanding and transfer learning prowess of pre-trained Transformers with the fine-grained, high-sensitivity motif detection capability of CNNs in a manner specifically optimized for the nuanced task of bacterial promoter recognition. This is particularly crucial for accurately identifying atypical or weakly conserved promoters. Furthermore, overarching challenges related to model interpretability, computational efficiency for large-scale use, and robust generalization across diverse σ-factor types remain areas requiring focused innovation.

This review underscores the necessity for a novel framework that not only bridges these complementary capabilities but also leverages the latest advances in pre-training, attention mechanisms, and feature fusion. The proposed DNABERT2-CAMP model is designed to meet this need directly, introducing a dedicated hybrid architecture with explicit, synergistic modules for global context encoding, local feature refinement via a novel CAMP module, and adaptive fusion—aiming to establish a new benchmark in accuracy, robustness, and biological relevance for promoter identification.

## 3. Materials and Methods

### 3.1. Dataset Construction

#### Data Sources and Processing

To construct a high-quality and balanced dataset for promoter recognition in *E. coli*, we integrated sequences from multiple sources, as detailed in Table 1. Positive samples (promoter sequences) were primarily sourced from RegulonDB (version 10.5) [19], covering σ70, σ24, σ32, σ38, and σ54 promoters, with a sequence length of 81 bp. In this study, promoters associated with different σ factors were treated as a unified positive class, following a general promoter recognition setting. Additional experimentally validated promoter sequences were obtained from the literature [18]. Negative samples (non-promoter sequences) were extracted from the coding regions and intergenic regions of the *E. coli* K-12 genome via NCBI [20]. All sequences were standardized to a uniform length of 81 base pairs to ensure model input consistency.

To mitigate redundancy, all sequences were processed using CD-HIT [21] with a sequence similarity threshold of 0.8. After filtering and deduplication, the final dataset comprised 8720 sequences, with 4360 positive and 4360 negative samples, achieving a 1:1 class balance, as detailed in Table 1.

The dataset was partitioned into training, validation, and test sets in an 8:1:1 ratio using stratified sampling [22] to maintain the proportional class distribution across the subsets. An independent test set of 256 promoter sequences from Li et al. [18] was reserved for external validation, with no overlap and a sequence similarity ≤ 0.8 relative to the training data.

### 3.2. Sequence Encoding

Each DNA sequence was tokenized using a 6-mer strategy to conform to the input requirements of DNABERT-2 [11]. Specifically, an 81 bp sequence was segmented into overlapping 6-nucleotide tokens (e.g., “ATGCTA”, “TGCTAC”, …), separated by spaces. The tokenized sequences were then processed by the DNABERT-2 tokenizer to generate input_ids and attention_mask tensors. The maximum sequence length was set to 128 tokens to accommodate padding and truncation, ensuring compatibility with DNABERT-2’s input requirements. This tokenization approach preserves local sequence context and aligns with the pre-trained vocabulary of DNABERT-2, enabling the effective use of its pre-trained embeddings.

### 3.3. Model Architecture

**Figure 1:**
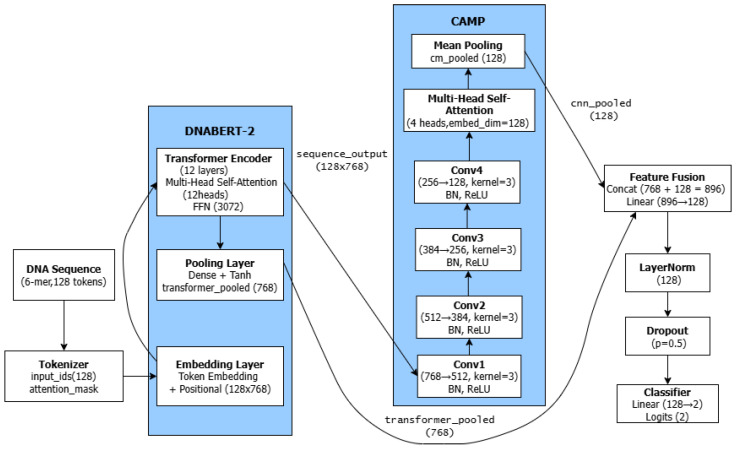
Schematic diagram of the DNABERT2-CAMP architecture showing the integration of DNABERT-2 and CAMP modules with feature fusion and classification components. Arrows indicate the forward flow of feature representations between different modules.

The proposed DNABERT2-CAMP model comprises two core components: (1) the DNABERT-2 feature extractor for global contextual modeling, and (2) the CAMP (CNN-Attention-Mean Pooling) module for local motif detection and feature refinement. As illustrated in Figure 1, these components work in a complementary manner to extract both global and local features from DNA sequences. The integration of Transformers for global context and CNNs for local patterns is an emerging and effective design in bioinformatics [23].

The demonstrated superiority of DNABERT2-CAMP stems from its effective hybrid architecture, which integrates the global contextual modeling power of DNABERT-2 with the local motif detection capability of CNNs, refined by a multi-head self-attention mechanism. The CAMP module plays a crucial role in bridging the gap between global contextual understanding and local motif detection. By sequentially applying CNN for local pattern extraction, multi-head attention for feature interaction modeling, and mean pooling for dimensionality reduction, the CAMP module generates biologically meaningful representations that complement the global embeddings from DNABERT-2.

### 3.4. DNABERT-2 Feature Extractor

We employed the pre-trained DNABERT-2 model [11] as the foundational feature extractor in our hybrid architecture. This selection is motivated by the model’s demonstrated efficacy in capturing global contextual information and long-range dependencies within DNA sequences—attributes critical for accurately identifying promoter regions, especially those involving conserved yet spatially separated motifs such as the −10 and −35 boxes in σ70-type promoters.

Leveraging a Transformer-based architecture pre-trained on large-scale genomic data via masked language modeling (MLM), DNABERT-2 utilizes 6-mer tokenization to segment DNA sequences into semantically meaningful units. This approach effectively preserves local nucleotide context while enabling the self-attention mechanism to model interactions across the entire sequence. Such a capability is particularly advantageous for recognizing non-canonical promoters and handling variable spacer lengths, which pose significant challenges to traditional methods, such as position-weight matrices (PWMs) and convolutional models.

In our implementation, we used the pre-trained DNABERT-2 model, which comprises 12 Transformer encoder layers, each with 12 self-attention heads and a hidden dimension of 768. The model processes tokenized input sequences and generates two forms of representations:

- A sequence-level pooled embedding(transformer_pooled, 768-dimensional) that encapsulates global sequence semantics.
- Token-wise contextual embeddings (sequence_output, dimensions: [batch_size, 128, 768]), which retain positional information, are subsequently passed to the CNN module for local feature refinement.

By integrating DNABERT-2, our model benefits from transfer learning and biological prior knowledge encoded during pre-training, thereby reducing reliance on manual feature engineering and enhancing generalization across diverse promoter classes.

The foundation of our model is the pre-trained DNABERT-2 Transformer. Unlike traditional encoding methods, DNABERT-2 provides rich, contextualized embeddings by processing DNA sequences using 6-mer tokenization across a large pre-training corpus [11]. This enables the model to effectively capture complex non-linear relationships and long-range dependencies within promoter regions.

### 3.5. CAMP Module: CNN, Multi-Head Attention, and Mean Pooling

The CAMP (CNN-Attention-Mean Pooling) module is designed to extract local sequence patterns and enhance feature representation through three sequential components:

#### 3.5.1. Convolutional Neural Network

We employ a four-layer 1D CNN with the following configuration:

- Conv1: 768 → 512 channels, kernel size = 3, padding = “same”
- Conv2: 512 → 384 channels, kernel size = 3, padding = “same”
- Conv3: 384 → 256 channels, kernel size = 3, padding = “same”
- Conv4: 256 → 128 channels, kernel size = 3, padding = “same”

Each convolutional layer is followed by batch normalization and ReLU activation. The progressive reduction in channel dimensions (768 → 512 → 384 → 256 → 128) enables hierarchical feature abstraction, effectively capturing conserved promoter motifs such as the −10 (TATAAT) and −35 (TTGACA) boxes while suppressing irrelevant background noise.

#### 3.5.2. Multi-Head Self-Attention

Following the CNN layers, we incorporate a multi-head self-attention mechanism with 4 attention heads (embedding dimension = 128). This component allows the model to dynamically weight the importance of different sequence regions and capture long-range dependencies between spatially separated regulatory elements, which is particularly crucial for recognizing the interaction between −10 and −35 boxes in *σ*70 promoters.

The attention mechanism computes:

(1) $$\mathrm{Attention}\left(Q,K,V\right)=\mathrm{softmax}\left(\frac{QK^{T}}{\sqrt{d_{k}}}\right)V$$

where *Q*, *K*, and *V* represent queries, keys, and values derived from the CNN output features, and $d_{k}$ is the dimension of the key vectors [24].

#### 3.5.3. Mean Pooling

The attention outputs undergo mean pooling along the sequence dimension, yielding a compact 128-dimensional feature vector (cnn_pooled) that encapsulates the most salient local features. This pooling operation is defined as:

(2) $$\mathrm{cnn}\_\mathrm{pooled}=\frac{1}{L}\sum_{i=1}^{L}h_{i}$$

where *L* is the sequence length and $h_{i}$ represents the hidden state at position *i* from the attention layer [25].

The CAMP module effectively bridges the gap between local motif detection and global sequence understanding, generating biologically meaningful representations that complement the contextual embeddings from DNABERT-2.

While DNABERT-2 excels at global understanding, the addition of the CNN and Multi-Head Attention layers is crucial for robust classification. The convolutional layers are specifically adept at capturing local, biologically relevant sequence motifs, such as the conserved −10 (Pribnow box) and −35 elements characteristic of σ70-dependent promoters [4,12]. By fusing the global contextual features from DNABERT-2 with these locally extracted motif features, the model achieves a more comprehensive and biologically grounded representation of the DNA sequence. Furthermore, the multi-head self-attention mechanism refines the overall feature space, enabling the model to dynamically weigh the importance of different sequence segments before the final classification, thereby boosting generalization and resistance to sequence noise [24].

### 3.6. Output Layer

The global representation from DNABERT-2 (transformer_pooled, 768-dim) and the local representation from the CAMP module (cnn_pooled, 128-dim) are concatenated into a combined 896-dimensional feature vector. This vector is then passed through a fully connected layer to reduce its dimensionality to 128, followed by layer normalization [26] and dropout (rate = 0.5) [27] for regularization. Finally, a linear classifier projects the 128-dimensional features to 2-dimensional logits for binary classification.

### 3.7. Model Training and Evaluation

#### 3.7.1. Training Strategy

The model was trained using a weighted cross-entropy loss function [28] to handle class imbalance, which is a common alternative to data-level approaches such as oversampling [29], with the AdamW optimizer [30] and a cosine learning rate scheduler. The initial learning rate was set to $2\times 10^{-5}$, with a batch size of 8 per device. Gradient accumulation (steps = 4) and clipping (max norm = 0.8) were applied to stabilize training. Early stopping was implemented with a patience of 10 epochs based on the validation ROC AUC [31].

We performed five-fold cross-validation [22] on the training set, with each fold containing approximately 5198 training and 1300 validation sequences. Model performance was evaluated using accuracy (ACC), sensitivity (Sn), specificity (Sp), precision, F1-score, Matthews correlation coefficient (MCC) [32] and ROC AUC [33]. The best model from each fold was selected based on validation performance and evaluated on both the internal test set (722 sequences) and the independent test set (256 sequences).

#### 3.7.2. Hyperparameter Optimization

To ensure optimal model performance, we conducted systematic hyperparameter tuning through multiple experiments, combining grid search with manual adjustments. Key parameters, such as learning rate, batch size, weight decay, and warmup steps, were optimized based on validation performance. The final hyperparameter values are summarized in Table 2.

### 3.8. Statistical Analysis

To assess the statistical significance of performance differences between DNABERT2-CAMP and DNABERT, we employed two complementary non-parametric tests.

For the five-fold cross-validation results, fold-wise AUC values were compared using the Wilcoxon signed-rank test. This test evaluates whether the median of paired differences significantly deviates from zero. Both two-sided and one-sided tests were performed, with the latter testing the directional hypothesis that DNABERT2-CAMP outperforms DNABERT.

For the independent test set, we applied DeLong’s test to compare ROC AUC values [34]. This method accounts for the paired nature of predictions on the same samples and provides a variance estimate for the difference in ROC AUC. We report the ROC AUC difference, 95% confidence interval, and the *p*-value.

All statistical analyses were conducted using Python (version 3.9.1; SciPy, scikit-learn) and R (version 4.3.0; pROC package). A *p*-value < 0.05 was considered statistically significant.

### 3.9. Code Availability

The implementation of DNABERT2-CAMP, along with preprocessing, training, and evaluation scripts, is freely available under an open-source license at: https://github.com/hualinxu/dnabert2_camp (accessed on 25 December 2025) This repository includes detailed instructions to reproduce the results reported in this paper.

## 4. Results and Discussion

### 4.1. Model Performance and Comparative Analysis

The performance of the DNABERT2-CAMP model was rigorously evaluated using five-fold cross-validation on the training and validation sets. As summarized in Table 3, the model achieved an accuracy (ACC) of 93.10% and a ROC AUC of 97.28%, alongside a balanced sensitivity (Sn) of 92.42% and specificity (Sp) of 93.00%. The high Matthews Correlation Coefficient (MCC) of 0.8604 further confirms the model’s robust and balanced classification capability [32].

To assess generalization, the model was evaluated on an internal test set (722 sequences) and an independent external test set (256 sequences). As detailed in Table 4, the model maintained strong performance on the internal test set (ACC: 91.90%, ROC AUC: 95.72%) and the independent test set (ACC: 89.83%, ROC AUC: 92.79%), demonstrating its effectiveness on unseen data.

A comprehensive comparison was conducted against eight existing promoter recognition models, encompassing traditional feature engineering methods (e.g., Z-curve [14], Stability [13]), classical machine learning (e.g., iPro54 [14]), early deep learning models (e.g., iPromoter-2L [16], MULTiPly [17], iPromoter-BnCNN [15]), a CNN with attention [18], and the Transformer-based DNABERT [11]. The results, presented in Table 5, unequivocally show that DNABERT2-CAMP outperforms all these methods across all key metrics. Specifically, it surpasses the previous state-of-the-art DNABERT model by +2.11% in accuracy and +1.41% in ROC AUC. The ROC AUC value indicates exceptional robustness in distinguishing promoter sequences from non-promoter sequences across different classification thresholds—a critical attribute in genomic applications where false positives and negatives can severely impact downstream research, including gene expression profile analysis and synthetic biology design [12].

**Figure 2:**
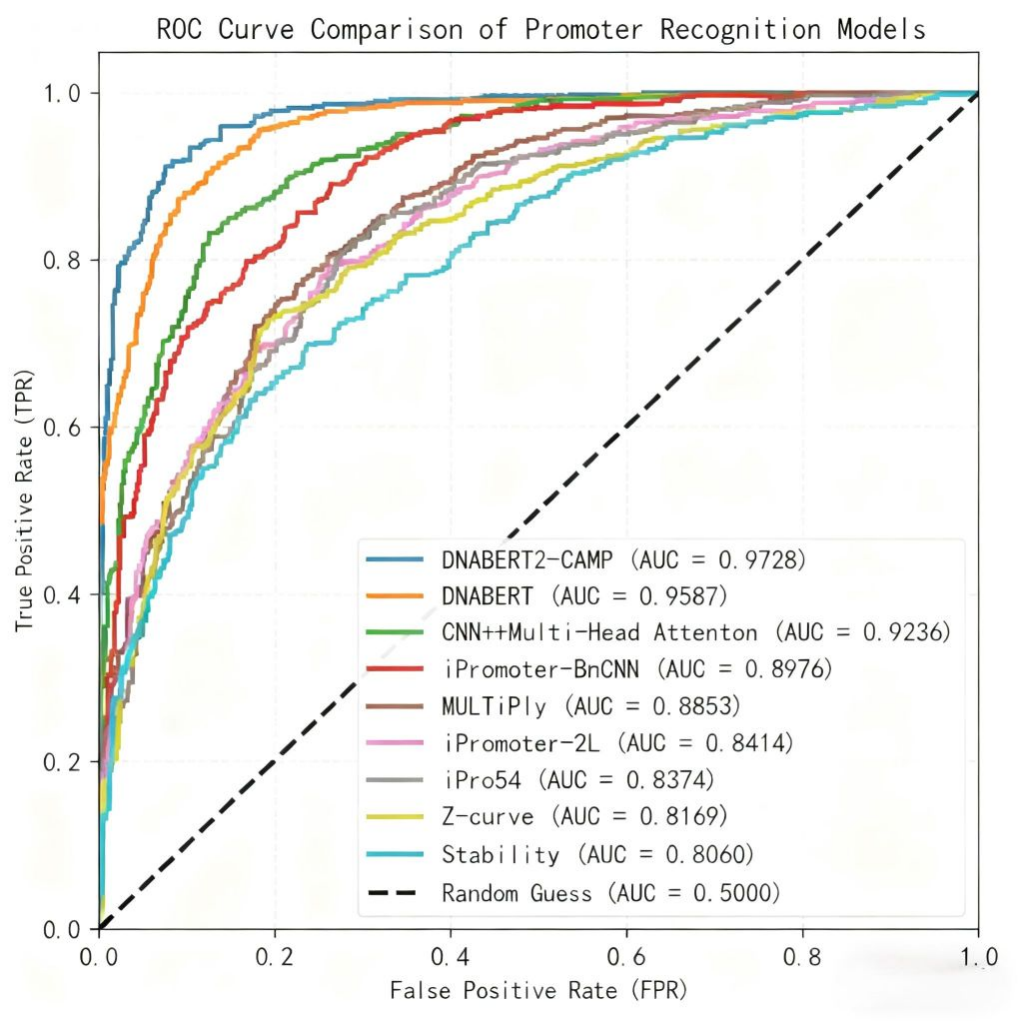
ROC Curve Comparison of Promoter Recognition Models.

Figure 2 illustrates the ROC curves of DNABERT2-CAMP and eight competing promoter recognition models, providing a visual comparison of their classification performance across varying decision thresholds. The ROC curve of DNABERT2-CAMP consistently occupies the top-left corner of the plot, indicating superior true positive rates at low false positive rates across all thresholds. This visual superiority aligns with the quantitative results presented in Table 5, where DNABERT2-CAMP achieved the highest ROC AUC (97.28%). Notably, the curve demonstrates a steep initial ascent, reflecting high sensitivity in detecting promoter sequences even under stringent specificity requirements—a critical characteristic for practical genomic applications. In contrast, traditional feature-based methods such as Z-curve and Stability exhibit flatter curves, indicating limited discriminative power. The clear separation between DNABERT2-CAMP and other deep learning models (e.g., DNABERT, iPromoter-BnCNN) further underscores the effectiveness of integrating global contextual embeddings with local motif detection and attention mechanisms.

### 4.2. Statistical Validation of Performance Improvements

The statistical significance of the performance improvement offered by DNABERT2-CAMP was assessed on both the cross-validation and independent test sets (Table 6).

In the five-fold cross-validation, the model achieved a mean ROC AUC of 0.9728, an increase of 0.0141 over DNABERT (0.9587). The Wilcoxon signed-rank test on the fold-wise AUCs yielded a one-sided *p*-value of 0.0313 and a two-sided *p*-value of 0.0625. On the independent test set, DNABERT2-CAMP achieved an ROC AUC of 0.9279, which was 0.0129 higher than that of DNABERT (0.9150). DeLong’s test confirmed that this improvement was statistically significant (p=0.004).

These results indicate that while the performance gain in cross-validation shows a directional trend, the superior generalization capability of DNABERT2-CAMP is unequivocally demonstrated by its statistically significant performance on the independent test set.

The superior performance of DNABERT2-CAMP can be attributed to its synergistic integration of global contextual embeddings from DNABERT-2 and localized motif detection via the CAMP module. While DNABERT-2 captures long-range dependencies and conserved promoter architecture across the entire 81 bp sequence, the CNN component effectively identifies fine-grained motifs such as the −10 and −35 boxes. The multi-head attention mechanism further refines these features by modeling distributed global contextual dependencies across the sequence, enabling the model to handle variable spacer lengths and degenerate motifs commonly observed in non-σ70 promoters. This hybrid approach addresses the limitations of standalone CNN models, which struggle with long-range interactions, and pure Transformer models, which may lack high-resolution motif sensitivity.

### 4.3. Impact of Optimization and Comparative Analysis with Existing Methods

The strong performance of DNABERT2-CAMP can also be attributed to its carefully optimized training strategy and hyperparameter tuning, as detailed in Section 3.7.2. The selected learning rate of $2\times 10^{-5}$ with cosine scheduling combined with gradient accumulation and clipping, ensured stable convergence for the complex hybrid architecture. The use of a weighted cross-entropy loss and early stopping further enhanced the model’s ability to handle potential class imbalance and prevent overfitting [28,31].

Our comparative analysis reveals the distinct advantages of this hybrid approach. Traditional methods like PWMs and feature engineering (e.g., Z-curve, Stability) are limited by their reliance on manual feature extraction and positional assumptions, making them ineffective for atypical promoters [6,13,14]. Machine learning techniques, like SVMs (e.g., iPro54 [14]) improve upon this but remain constrained by the quality of feature engineering and an inability to model long-range dependencies [35]. While CNNs (e.g., MULTiPly [17], iPromoter-BnCNN [15]) excel at local motif detection, they struggle with long-range context, and RNNs face challenges with computational costs and vanishing gradients [9,35]. Transformer-based models like DNABERT [11] capture global context effectively but have inferior local feature extraction accuracy compared to CNNs [8]. DNABERT2-CAMP successfully addresses these complementary limitations through its unique design, explaining its state-of-the-art performance.

### 4.4. Analysis of Model Decisions and Biological Basis

While the proposed DNABERT2-CAMP model demonstrates strong predictive performance across multiple evaluation settings, understanding the biological rationale behind its decisions is essential for assessing its reliability and practical applicability. In this section, we further analyze the internal behavior of the hybrid model from both global contextual and local motif perspectives, focusing on the Transformer attention mechanism and CNN activation patterns.

#### 4.4.1. Attention-Based Interpretability Analysis

To investigate how the Transformer encoder processes promoter sequences, we analyzed the attention distributions of the DNABERT-2 component on the test dataset. All sequences used in this analysis were derived from the curated *E. coli* promoter dataset described in Section 3.1, comprising 8720 samples with an original length of 81 bp. Only attention scores corresponding to the original 81 bp sequence region were retained for analysis.

**Figure 3:**
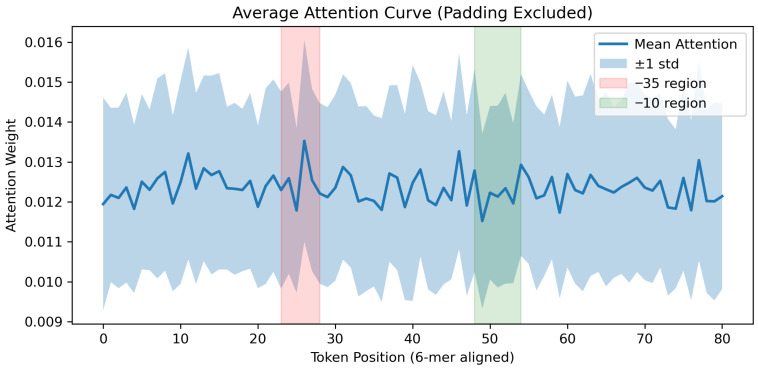
Average attention distribution across token positions for all test samples. Attention scores are averaged across all attention heads in the last Transformer layer. The solid line represents the mean attention value at each token position, while the shaded area indicates the corresponding standard deviation, demonstrating stable and distributed contextual representations learned by the DNABERT-2 encoder.

As shown in Figure 3, the attention distribution exhibits a smooth profile along the sequence, with relatively small variance across samples. The attention visualization is not intended to be a direct indicator of motif localization, but rather reflects distributed global contextual encoding. This indicates that the model captures distributed contextual information rather than relying excessively on isolated positions. Notably, the canonical −35 and −10 promoter regions are located within consistently attended segments of the sequence, rather than appearing as isolated attention peaks. Such behavior is biologically reasonable, as promoter recognition typically depends on the cooperative contribution of multiple sequence regions rather than a single nucleotide position.

We further compared the attention patterns between positive (promoter) and negative samples. The overall attention trends of the two groups remain largely consistent, while subtle local differences can be observed in specific regions. This suggests that the Transformer encoder focuses on comparable contextual regions for both classes and that the final discrimination is not solely driven by attention differences, but by subsequent feature integration. Overall, these results demonstrate that the attention mechanism learns stable and biologically plausible contextual representations, supporting the robustness of the model’s decision process.

#### 4.4.2. CNN Motif Activation and Peak Analysis

Precise local promoter motif recognition, such as the −10 and −35 elements, is mainly handled by the CNN component in the CAMP module. To investigate the local sequence patterns learned by the CNN component, we analyzed the sequence regions corresponding to the top 10% highest convolutional activation values in the same test dataset used in Section 4.4.1 (8720 *E. coli* promoter sequences). From these highly activated regions, short sequence motifs were extracted and ranked according to their occurrence frequency. Representative motifs include AAAAAA, TAAAAA, AAAAAT, TTTTTT, and ATAAAA, indicating that the majority of high-activation patterns are strongly enriched in adenine and thymine nucleotides. This observation suggests that the CNN module predominantly captures AT-rich sequence characteristics.

This AT-rich enrichment is biologically consistent with the known properties of bacterial *σ*70 promoters. In particular, the canonical −10 promoter element (TATAAT) is characterized by high AT content and substantial degeneracy across natural *E. coli* promoters. Rather than reproducing the exact consensus sequence, the CNN learns flexible and degenerate variants of the −10 box, reflecting the compositional bias and structural tolerance observed in experimentally validated promoter regions.

In addition to AT-rich motifs, several GC-containing patterns were also identified among the highly activated regions, including TGCTGA, CTGGCA, GCTGGC, and CTGGCG. These motifs partially resemble the TTG/CTG core elements of the canonical −35 box (TTGACA), which is known to exhibit greater sequence variability compared to the −10 region. The presence of these GC-enriched motifs suggests that the CNN component captures a broader spectrum of promoter-associated sequence features, including degenerate −35-like elements and adjacent regulatory contexts.

To further assess the stability and reliability of the learned convolutional features, we analyzed the peak activation statistics of the CNN module across promoter samples. The activation peaks exhibited a consistent magnitude distribution, with an average peak value of approximately 0.11 and a low relative standard deviation (around 2%). Moreover, the maximum activation values remained within a narrow range across samples, indicating that no individual sequence position or spurious pattern dominated the convolutional responses. This stability suggests that the CNN module consistently responds to informative local sequence patterns rather than random noise or isolated outliers.

Overall, the motif composition and peak activation analyses jointly demonstrate that the CNN component learns biologically meaningful and stable representations of promoter-associated motifs. When integrated with the global contextual embeddings produced by the DNABERT-2 Transformer, these locally refined features contribute to robust and interpretable promoter discrimination while remaining consistent with the established architecture of bacterial promoter regions.

### 4.5. Computational Considerations, Limitations, and Future Directions

The high accuracy of DNABERT2-CAMP on σ70 promoters demonstrates its strength in learning strong consensus motifs. However, its performance on non-σ70 promoters, while robust given the data scarcity (see Table 1), indicates an area for growth that reflects a fundamental biological challenge: the motifs for alternative σ factors are more degenerate and less stereotyped. This performance pattern itself is biologically informative, underscoring that accurate recognition of diverse promoter classes is contingent upon both model architecture and the availability of high-quality training data that captures biological variation. Future efforts to curate more balanced datasets will directly enhance the model’s ability to generalize across the natural diversity of transcriptional regulatory sequences.

Several limitations warrant attention. The model’s performance on non-*σ*70-type promoters is constrained by the scarcity of labeled data. Therefore, *σ*-factor specific promoter classification was not addressed in the current study and is identified as an important direction for future work when more balanced datasets become available. Furthermore, its reliance on large-scale pre-training data may pose challenges for application in rare microorganisms with limited genomic data.

Future research will focus on several promising directions:

(a) Cross-species application: Leveraging the “species-agnostic” 6-mer tokenization and pre-trained embeddings to extend the model’s application to cross-species promoter prediction.
(b) Enhanced interpretability: Utilizing techniques such as SHAP value analysis and saliency maps to uncover novel biological insights into promoter architecture [36].
(c) Addressing data scarcity: Exploring data augmentation, transfer learning, and few-shot learning strategies for rare promoter classes.
(d) Model efficiency: Investigating model compression techniques to facilitate deployment in high-throughput synthetic biology pipelines.
(e) Multi-task learning: Developing frameworks to simultaneously predict promoter strength, transcription factor binding sites, and other regulatory elements for a more comprehensive view of gene regulation [37].

Ultimately, these advancements will transition DNABERT2-CAMP from a predictive tool into a fundamental discovery platform, deepening our understanding of gene regulatory networks and providing the technical foundation for rational design in synthetic biology.

## 5. Conclusions

In this study, we proposed DNABERT2-CAMP, a hybrid deep learning framework that synergistically integrates the global contextual modeling capabilities of DNABERT-2 with the local motif detection strength of convolutional neural networks, augmented by a multi-head self-attention mechanism. Comprehensive evaluations demonstrated that DNABERT2-CAMP consistently outperforms existing state-of-the-art methods, achieving a ROC AUC of 97.28% and an accuracy of 93.10% in five-fold cross-validation while maintaining robust generalization on independent test sets (ROC AUC: 92.79%, accuracy: 89.83%).

Beyond methodological innovation, this work provides significant biological insights and practical value. The hybrid architecture effectively bridges the long-standing gap between global sequence context understanding and precise local motif detection—a critical challenge in promoter recognition. By fusing transfer-learned semantic embeddings from DNABERT-2 with CNN-extracted motif features, our model captures both the conserved −10/−35 boxes of σ70 promoters and the diverse patterns of non-σ70 promoters, offering enhanced biological interpretability. This capability is particularly valuable for synthetic biology applications, where accurate promoter identification enables rational design of genetic circuits, biosensors, and engineered microbial systems.

Despite these advances, several challenges remain. First, the model’s performance on non-σ70 promoters is constrained by dataset scarcity, highlighting the need for more balanced, multi-σ-factor training data. Second, computational efficiency requires optimization for genome-scale deployment; future work will explore model compression and lightweight architectures. Third, enhanced interpretability methods, such as attention visualization and feature importance analysis, could yield novel biological insights into promoter architecture.

Looking forward, we envision several promising research directions: (a) extending DNABERT2-CAMP to cross-species promoter prediction using its species-agnostic 6-mer tokenization; (b) integrating multi-task learning for simultaneous prediction of promoter strength, transcription factor binding sites, and other regulatory elements; (c) developing an end-to-end synthetic promoter design pipeline that combines our recognition model with generative approaches; and (d) validating model predictions through experimental assays to establish a closed-loop design-build-test-learn framework.

In summary, DNABERT2-CAMP establishes a new benchmark for accurate, robust, and interpretable promoter recognition in *E. coli*. By effectively combining the strengths of Transformers and CNNs, our framework not only advances computational genomics methodology but also provides a powerful tool for synthetic biology, gene regulation studies, and future genomic discovery. As high-throughput sequencing and synthetic biology continue to evolve, such integrative deep learning approaches will play an increasingly vital role in deciphering the regulatory code of life.

## Figures and Tables

**Table 1:** Dataset composition and distribution across different σ factor types.

Data Source	σ70	Non-σ70	Negative	Total
Literature [7]	1500	-	2860	4360
RegulonDB	1860	1000	-	2860
NCBI	-	-	1500	1500
Total	3360	1000	4360	8720

**Table 2:** Hyperparameter tuning ranges and final selected values for DNABERT2-CAMP.

Hyperparameter	Tuning Range	Final Value
Learning Rate	$5\times 10^{-6}$–$5\times 10^{-5}$	$2\times 10^{-5}$
Batch Size (Training)	4, 8, 16	8
Batch Size (Evaluation)	4, 8, 16	4
Gradient Accumulation Steps	2, 4, 8	4
Weight Decay	0.005–0.1	0.05
Warmup Steps	100–500	300
Learning Rate Scheduler	cosine, linear	cosine
Max Gradient Norm	0.6–1.0	0.8
Training Epochs	5–20	15
Early Stopping Patience	3–15	10
Early Stopping Threshold	0.1–0.3	0.1
Random Seed	42, 123	42

**Table 3:** Performance Metrics from Five-Fold Cross-Validation.

Evaluation Metric	Value
Accuracy	0.9310
Precision	0.9439
Recall	0.9242
F1 Score	0.9288
ROC AUC	0.9728
MCC	0.8604
Specificity	0.9300

**Table 4:** Performance Metrics on Test Set and Independent Test Set.

Evaluation Metric	Internal Test Set	Independent Test Set
Accuracy	0.9190	0.8983
Precision	0.9253	0.9078
Recall	0.9146	0.8903
F1 Score	0.9162	0.8962
ROC AUC	0.9572	0.9279
MCC	0.8359	0.8014
Specificity	0.9183	0.8958

**Table 5:** Comparison of Recognition Performance Among DNABERT2-CAMP and Existing Models Using Five-Fold Cross-Validation.

Model	ACC (%)	Sn (%)	Sp (%)	ROC AUC (%)	MCC
Z-curve [14]	80.33	76.68	82.50	81.69	0.5980
Stability [13]	77.96	75.95	78.64	80.60	0.5613
iPro54 [14]	80.47	76.83	82.71	83.74	0.5983
iPromoter-2L [16]	81.74	79.23	83.58	84.14	0.6327
MULTiPly [17]	86.93	87.26	86.20	88.53	0.7368
iPromoter-BnCNN [15]	88.16	88.73	87.24	89.76	0.7549
CNN + Multi-Head Attention [18]	90.24	90.35	90.13	92.36	0.8016
DNABERT [11]	90.99	88.97	89.12	95.87	0.8255
DNABERT2-CAMP	93.10	92.42	93.00	97.28	0.8604

**Table 6:** Statistical comparison between DNABERT2-CAMP and DNABERT.

Dataset/Metric	DNABERT2-CAMP	DNABERT	Test	*p*-Value
Cross-validation (5-fold AUC)	0.9728	0.9587	Wilcoxon signed-rank	0.0625 (two-sided), 0.0313 (one-sided)
Independent test set (AUC)	0.9279	0.9150	DeLong test	0.004

## Data Availability

The source code for the DNABERT2-CAMP model, the curated dataset of 8720 sequences (4360 positive and 4360 negative samples), and all scripts necessary to reproduce the findings of this study are publicly available in the GitHub repository at https://github.com/hualinxu/dnabert2_camp (accessed on 25 December 2025). The exact version of the software and data used in this study has been archived and can be cited as Release v1.0. The original promoter sequences were sourced from RegulonDB (version 10.5) [19] and the literature [18], while the non-promoter sequences were extracted from the *E. coli* K-12 genome available from NCBI [20].
